# Using the Exploratory Sequential Mixed Methods Design to Investigate Dental Patients’ Perceptions and Needs Concerning Oral Cancer Information, Examination, Prevention and Behavior

**DOI:** 10.3390/ijerph18147562

**Published:** 2021-07-16

**Authors:** Mohammed Jafer, Rik Crutzen, Abdalla Ibrahim, Ibtisam Moafa, Haitham Zaylaee, Mohammad Ajeely, Bart van den Borne, Alessio Zanza, Luca Testarelli, Shankargouda Patil

**Affiliations:** 1 Department of Preventive Dental Science, College of Dentistry, Jazan University, Jazan 45142, Saudi Arabia; ibtisam.moafa@gmail.com; 2 Department of Health Promotion, Maastricht University/CAPHRI, 6200 MD Maastricht, The Netherlands; Rik.Crutzen@maastrichtuniversity.nl (R.C.); abdalla.ibrahim@gmail.com (A.I.); b.vdborne@maastrichtuniversity.nl (B.v.d.B.); 3 Faculty of Dentistry, Jazan University, Jazan 45142, Saudi Arabia; haitham.zaylaee@gmail.com (H.Z.); mdical16@gmail.com (M.A.); 4 Department of Oral and Maxillofacial Sciences, Sapienza University of Rome, 00185 Rome, Italy; ale.zanza@gmail.com (A.Z.); luca.testarelli@uniroma1.it (L.T.); 5 Department of Maxillofacial Surgery and Diagnostic Sciences, Division of Oral Pathology, College of Dentistry, Jazan University, Jazan 45142, Saudi Arabia; dr.ravipatil@gmail.com

**Keywords:** early detection, mixed methods design, oral cancer, patient education, qualitative study, risk factors

## Abstract

Objectives: The objective of this study was to investigate dental patients’ behavior, thoughts, opinions and needs for oral cancer information, and dentists’ behavior regarding prevention and examination of oral cancer. Materials and Methods: This study utilized an exploratory sequential mixed methods design. Semi-structured interviews with open-ended questions were conducted for forty dental patients of both sexes. Based on the qualitative analysis, a structured questionnaire was developed and distributed among the participants. Data were analyzed for 315 participants to quantify their thoughts, needs, behavior and behavior expected from dentists regarding oral cancer. Frequency, percentages and cumulative percentages were calculated. Results: This study reveals that patients’ oral cancer knowledge levels were adequate, but most reported that their dentist had never examined them for oral cancer. Additionally, the participants had never performed self-examinations for oral cancer, nor were they aware of the possibility of doing so. Participants showed a preference for being examined and educated by their dentist about oral cancer and believed it would help early detection. Conclusions: The study participants are aware of oral cancer and its risk factors. The practice of oral cancer examinations and patient education of its risk factors by dental practitioners is limited. Patients feel a need for more attention to be paid to oral cancer examinations, preventive measures and targeted information on oral cancer risk factors.

## 1. Introduction

Oral cancer is one of the most prevalent cancers in the world [1]. In Saudi Arabia, oral cancer, together with head and neck cancer, is the most prevalent form of the disease, according to the latest Saudi Cancer Registry [2]. In particular, the Jazan region of Saudi Arabia has the highest rate of oral cancer, as indicated by registrations since 1999 [2,3,4]. Oral cancer is one of the most aggressive forms of cancer, and it has a low survival rate [5,6]. The survival rate of oral cancer is negatively affected by its tendency towards a late first diagnosis time [5,6,7,8,9]. Tobacco is a significant risk factor for oral cancer, particularly with smokeless forms of the substance such as gutkha, khaini and shammah [10]. Smokeless tobacco (ST) is frequently mixed with other substances and is either inhaled by sniffing, chewed or placed in the mouth against the mucosa sites [11]. The use of ST is prevalent globally, with many different recipes found worldwide [12,13,14]. In Saudi Arabia, the main ST product used is shammah, which was found to be the major independent risk factor for oral cancer in the Jazan region [15]. Data on the extent of shammah use in Saudi Arabia are absent. However, most diagnosed oral cancer patients are from the Jazan region of Saudi Arabia and have reported using shammah [15,16].

A lack of public awareness about oral cancer signs and symptoms and oral cancer self-examination also influences the odds of being diagnosed [17,18]. Early stages of oral cancer are usually asymptomatic, thereby affecting patients’ ability to recognize symptoms and leading to a delay in oral cancer diagnosis [9]. Moreover, if noticed, the signs and symptoms of oral cancer are usually misattributed to other manifestations of dental diseases, such as a common infection [19]. In addition to the silent nature of oral cancer, the routine practice of oral cancer examinations, which is associated with a better prognosis of the disease [20], is usually not practiced by dentists in countries such as the UK [21]. This lack of routine checkup for oral cancers is also prevalent among dentists in the Jazan region of Saudi Arabia [22,23]. Therefore, by utilizing an exploratory sequential mixed methods design (three-phase procedure), the primary objectives of the present study were as follows: to investigate the thoughts, opinions and needs for oral cancer information and dentists’ behavior regarding prevention and examination of oral cancer, as well as patients’ behavior regarding oral cancer (qualitative phase) among dental patients visiting the Jazan Dental School (JDS).

## 2. Materials and Methods

This study was performed in line with the principles of the Declaration of Helsinki. Approval was granted by the Ethics Committee of Jazan University registry no. [CDREC-06], dated 21 December 2016, prior to conducting the present study.

### 2.1. Qualitative Method

Semi-structured interviews with mainly open-ended questions were conducted with dental patients of both sexes in a one-on-one interview. The interview guide contained 14 open-ended questions and was created by the authors. The questions focused on patients’ knowledge of oral cancer, perceived risks, the seriousness of oral cancer and patients’ experiences and perceptions of the feasibility of oral cancer examinations. It also explored preferences for oral cancer examinations, education about oral cancer and its risk factors in the dental clinic and patients’ self-examination. Before use, the interview guide was evaluated by two experts in health promotion and oral cancer from JDS.

The interview guide was pre-tested with four volunteer patients of different educational levels from JDS clinics to evaluate the appropriateness of the language and guiding questions. The reporting of the qualitative study was in line with the Consolidated Criteria for Reporting Qualitative research (COREQ) checklist [24]. All of the interviews were conducted by a male and a female dental public health specialist from JDS, Saudi Arabia. To ensure consistency during the interviews, both interviewers conducted three mock interviews with individuals of the same gender from the targeted group, under direct supervision from a Professor of Health Promotion from Jazan University.

#### 2.1.1. Qualitative Participant Selection

All patients visiting JDS male and female clinics from 1 March to 19 April 2017 were invited for an interview and were informed about its purpose. A maximum of three individual interviews per day were carried out by an interviewer. Forty participants (21 females and 19 males) in the age range of 23 to 60 years were included in the study. The participants had different educational backgrounds, with the majority holding secondary school-level education. The majority of the participants were Saudi nationals (*n* = 35), with four non-Saudi female participants and one non-Saudi male participant.

#### 2.1.2. Qualitative Data Collection Procedure

All interviews were conducted in the Arabic language. Each interview lasted about 60 min. Interviewers took notes for all the interviews; however, only male interviews were audio recorded. The female participants were not recorded due to their personal preferences.

#### 2.1.3. Qualitative Data Analysis

A grounded theory methodology [25] was utilized to discover the emerging patterns in the patients’ thoughts, opinions and expectations regarding oral cancer and its related aspects. After each interview, the notes were reviewed with the participant to check if they represented their thoughts and opinions. For example, some participants mentioned that their dentist had examined them for oral cancer; however, they later indicated that they were not informed on oral cancer examinations. Additionally, all interview notes and audio recordings were reviewed by both interviewers together on the same day of the interview. Verbatim transcriptions and analyses were carried out in the Arabic language to avoid distortions in the data. The analysis was conducted manually, as there was no software program available that supported the Arabic language. Without referring to any participants, responses from all the interviews were grouped, and all similar responses were coded. Subsequently, focus codes were developed, which were later developed into theoretical codes (see Supplementary Materials File S1). The codes were reviewed and then translated to English by the third author, who was fluent in both languages. The English translations of the codes were reviewed and approved by both interviewers. Consequently, all codes were reported in the result section, along with the related participants’ responses. The quotations in the result section were mainly obtained from the male audio recordings; however, when looking at the notes, both the males’ and females’ interview notes were comparable.

### 2.2. Quantitative Method

#### 2.2.1. Quantitative Design

Following the qualitative study, a descriptive quantitative observational study was conducted to investigate and quantify oral cancer-related aspects among a larger sample of dental patients. Data were collected using a structured questionnaire from patients.

#### 2.2.2. Quantitative Participants and Procedure

This study was conducted in two JDS clinics, both for males and females, of Jazan University in Saudi Arabia. The clinics’ receptionists invited all regular dental patients who attended JDS clinics between 25 September and 31 December 2017 and who had at least one previous visit for a dental check-up to participate in this study.

Among all regular patients, 336 consented and returned the data collection forms to the JDS clinic receptions. Participation was voluntary, and no incentive was provided. Of those 336 participants, 21 did not complete the data collection forms and were not included in the data analysis, resulting in a final sample of 315 participants.

#### 2.2.3. Quantitative Measurement Instrument and Pre-Testing

A questionnaire was developed based on findings from the prior qualitative study, which explored the view of dental patients from JDS clinics regarding oral cancer and its related aspects. The questionnaire consisted of 55 items that incorporated the three main themes that arose from the qualitative study. The first part of the questionnaire assessed the participants’ knowledge of oral cancer etiology, the local epidemiology, risk factors for the disease and prevention strategies. The second part investigated the participants’ perceptions regarding oral cancer detection, and the role of clinical examinations, self-examination and preventive methods. It also included the perceived dentist roles regarding patient education and tobacco cessation. The final part evaluated the participants’ clinical experiences of oral cancer examination with their dentists, dentists’ practice of patient education and participants’ self-examination. The questionnaire was developed in English, translated into Arabic by an Arabic- and English-speaking dental public health specialist and reviewed and evaluated using cross-translation by the principal investigator.

The questionnaire was pre-tested among 15 male and 11 female returning JSD dental patients, using a thinking-aloud method, leading to a substantial revision to improve the participants’ understanding of several questions. Therefore, a re-evaluation of the modified questionnaire, using the thinking-aloud method, was carried out among 12 males and 13 females who were regular patients at JSD clinics. It was then sent to an Arabic-speaking dental public health specialist and two Arabic-speaking health promotion specialists for evaluation. They had no further concerns regarding the questionnaire.

#### 2.2.4. Quantitative Data Analysis

Descriptive statistics were calculated for the demographic variables. Frequencies and percentages were obtained for the individual items of the questionnaire. Furthermore, cumulative percentages of dental patients’ perceptions toward oral cancer examinations were calculated for item numbers 22, 23, 33, 34 and 35. Other cumulative percentages were obtained for dental patients’ clinical experiences, included in items 45, 51 and 52, which were considered if the participants were examined for oral cancer, informed of oral cancer examinations and educated about oral cancer and its risk factors.

## 3. Results

### 3.1. Qualitative Results

The analysis of the one-on-one interviews in the present study showed three major themes that represent the participants’ thoughts, opinions and behavior regarding oral cancer and its related aspects. The first central theme was the knowledge of the participants regarding oral cancer and its associated aspects. The second major theme was related to the participants’ perception of oral cancer and its related aspects. The third theme concerned the behavior (practice) of the participants and their dentists regarding oral cancer self-examination and clinical procedures.

### 3.2. Knowledge

The participants’ knowledge regarding oral cancer and its related aspects was one of the major themes that emerged from the analysis of the present study’s data. It was developed out of four subthemes: etiology, epidemiology, risk factors and prevention. Participants referred to oral cancer as the cancer of the mouth but did not know about its etiology. Several participants indicated that they had no idea of what oral cancer could mean, “I have never heard of cancer in the mouth.” Other participants thought that oral cancer could be the result of some type of bacterial or fungal infection, “my grandmother had an infection in her mouth that she neglected, and it became cancer… she died of it”.

Regarding the high prevalence of oral cancer in the Jazan region, almost half of the participants confirmed that the Jazan region has a high prevalence of oral cancer: “I have read in the media that the highest number of oral cancer patients was from the Jazan region.” However, many participants mentioned that they were not aware of its epidemiology: “I work in the health sector and had never heard of such a claim.” Other participants even argued that oral cancer is not prevalent in the region of Jazan, “I do not have oral cancer, nor does anyone of my family or my friends or their family”.

Most of the participants indicated that they did not know the risk factors of oral cancer. Though many thought it could be related to smoking, ghat chewing and shammah use, one participant stated, “I heard that one lady in the neighboring village died of cancer because she used Shamma.” Several other participants mentioned that smoking could be a risk factor; however, they believed it could be a risk factor in combination with other factors such as bacteria, alcohol, carelessness of the individual or poor oral hygiene. They thought that a local factor would have to be involved for smoking to cause harm in the mouth, “I do not believe that smoking alone can cause cancer in the mouth, maybe patients were using something else with it, like Ghat.” Other participants believed that it related to the individuals’ genetics or their environment, “my dad used to smoke and chew Ghat for more than forty years and did not have cancer”; “the environment is the reason of all cancer.” Another participant mentioned that oral cancer could be because an individual had another type of cancer somewhere else in his or her body, “my friend had cancer in his leg bone, but then it spread to all of his body and finally reached his mouth”.

Regarding preventing oral cancer, several participants were not aware of the preventive measures they could take to avoid oral cancer. Other participants thought that regular dental check-ups could prevent oral cancer, “my dentist will let me know if he notices any issues in my mouth and will help me to solve it”; “if my dentist tells me there is a problem in my mouth that could become cancer, I will stop smoking”. Others mentioned that by maintaining good oral hygiene, an individual could prevent oral cancer, “I believe maintaining good oral hygiene is the key to preventing all oral health issues”. On the other hand, to prevent oral cancer, several participants thought that, in addition to good oral hygiene, an individual must not smoke, “I believe in preventing any cancer, I must not smoke”.

### 3.3. Perception

Several participants had negative perceptions towards oral cancer facilities and accessibility in the Jazan region. Several participants indicated that there is no facility in the Jazan region to detect oral cancer. Other participants mentioned that they were not aware of any facility in the Jazan region that could help in detecting oral cancer, “I am from Jazan, and if God wills that I have oral cancer, I do not know any facility in Jazan that could help me in detecting or later treating my problem”. However, other participants stated that oral cancer can be detected in the Jazan region, “in Jazan, we have several dental clinics and a hospital that can detect oral cancer”. Moreover, a participant declared that it is possible to have oral cancer detected in the Jazan region in several facilities but that it would be difficult to access them, “I believe that oral cancer could be diagnosed in some specialized hospital in Jazan, but the issue is the difficulties individuals may face getting to those hospitals”.

Regarding the benefits of having a routine clinical oral cancer examination, many participants thought it would be useful and would help detect oral cancer. However, several participants stated that, even though clinical examinations of oral cancer would help towards early detection of the disease, it would cause fear to be on the receiving end, “I believe oral cancer screening by a dentist will be very helpful but scary at the same time”; “I will be scared if my dentist tells me, he will examine me for oral cancer, even though I think it is good for me”. Similarly, many participants indicated that it would be helpful if dentists educated their patients about oral cancer.

### 3.4. Practice

Many participants believed that their dentists did not examine them for oral cancer, “I am absolutely sure that no dentists I have ever visited examined me for oral cancer, they only look for what I was complaining of”. Moreover, several others thought they were not examined for oral cancer by their dentists, “I am a heavy smoker, and I do not think any dentists examined me for oral cancer; they have not told me anything”. However, some participants thought that their dentist had not examined them for oral cancer because there would be no need for an oral cancer examination, “my dentist did not examine me for oral cancer because I did not ask him, and maybe because I do not have oral cancer symptoms”; “no dentist examined me for oral cancer and maybe because oral cancer is not widespread in Jazan”.

Similarly, many participants indicated that they had not received any health education regarding oral cancer from their dentists, “my dentist knew I am a heavy smoker and I chew Ghat, but he never educated me of oral cancer and its risk factors”.

Regarding self-examination for oral cancer, only one participant mentioned that she performs a regular self-check-up. Furthermore, many participants indicated that they did not practice oral cancer self-examination because they did not know how to do so, “I have not heard of this oral cancer self-examination before today”; “I do not know what oral cancer self-examination is, and if I knew I would have done it”; “I have several problems with my teeth and my mouth, and I usually look at my mouth, but I did not know that I could examine myself for oral cancer”.

### 3.5. Quantitative Results

The total number of participants who completed the questionnaires and consented to participation was 315. Of those, 290 participants reported their ages. The mean participant age was 31 ± 11 years (range of 12–70), with a mode of 25 years. Among the 313 participants who reported their sex, 41.2% were males, and 58.8% were females. The majority of participants who reported their nationality were Saudis (85.9%). Participants reported their levels of education as follows: 4.4% were uneducated, 7.9% had primary education, 15.6% had intermediate education, 24.1% had secondary education and 47.2% had university education (Table 1).

The first part of the questionnaire assessed the participants’ knowledge of oral cancer etiology, local epidemiology, risk factors of the disease and prevention strategies (Table 2). The majority of the participants provided correct responses to the knowledge items relating to the nature of the disease (items 1, 2 and 3). In addition, most participants provided correct answers to the questions relating to signs of oral cancer (items 4, 7 and 8). However, many did not know that swellings and lumps anywhere in or around the mouth can be signs of oral cancer (items 5 and 6) (Table 2a. Around 70% of the participants were aware of the prevalence of oral cancer in Saudi Arabia (item 9). However, most participants were not aware of the relatively high prevalence of oral cancer in the Jazan region (items 10 and 11) (Table 2b). Between 60 and 82% of participants knew about the main risk factors for oral cancer (Table 2c). Moreover, most participants had correct knowledge of preventative measures for oral cancer, except for the effects of physical activity (item 18) (Table 2d).

The second part of the questionnaire investigated the participants’ perceptions regarding oral cancer detection, the role of clinical examinations and preventative measures (Table 3). The majority of participants responded favorably towards oral cancer detection (Table 3a). Furthermore, most participants believed that oral cancer examination should be routine in dental clinics (Table 3b). Slightly more than half of the participants believed that dentists often skip oral cancer examination because they only focus on patients’ dental complaints (item 26). In addition, the majority of participants said that the clinics (dental clinic or hospital), where the dentist meets the patient, impact the likelihood of dentists performing oral cancer examinations (item 32). Almost half of the participants mentioned that they would feel uncomfortable and uncertain when being examined for oral cancer (items 33 and 35). Nevertheless, slightly more than half of the participants expressed positive feelings about being examined for oral cancer (item 34). The majority of participants had favorable attitudes toward different oral cancer preventative measures, such as health education, community lectures and symposiums and distributions of written educational material about oral cancer (items 36, 37, 38, 39). Around three quarters of the participants favored banning ghat, shammah and tobacco to prevent oral cancer in the community (item 40; Table 3c).

The final part of the questionnaire assessed oral cancer practices concerning oral cancer detection, oral cancer clinical examination by dentists, oral cancer clinical education, oral cancer self-examination and the perceived role of dentists (see Table 4). A vast majority of the participants reported that their dentist never (62.3%) or rarely (14.1%) examined them for oral cancer (Table 4b). The reasons participants mentioned for not being examined for oral cancer by their dentist were because they said they did not have any signs or symptoms of oral cancer and because they had visited the dentist for a specific dental issue (items 41 and 43; Table 4a). Additionally, most participants showed a positive intention toward visiting the dentist when noticing changes that might indicate signs of oral cancer (Table 4b). The majority of participants also reported that their dentist had not educated them about oral cancer and its risk factors (Table 4c).

Furthermore, the majority of the participants said that they do not perform oral cancer self-examinations (Table 4d). Finally, a large majority of participants favored dentists having a role in terms of patient education about oral cancer and referrals for tobacco cessation programs, if needed (Table 4e). Although dentists’ examinations for oral cancer caused fear and uncertainty among dental patients (items 33 through 35), a high percentage of dental patients are in favor of routine oral cancer examinations by dental clinic health workers (items 22 and 23).

## 4. Discussion

The first study explored aspects possibly related to oral cancer, as perceived by dental patients who visit JDS clinics. Three major themes resulted from the participants’ narratives: oral cancer knowledge, perceptions of oral cancer examination and the practice of oral cancer examination. Delays in oral cancer detection would reduce the oral cancer five-year survival rate [5]. At an advanced stage of oral cancer, the tumor will have metastasized to local lymph nodes, thereby necessitating an invasive procedure that is usually extensive and will likely result in disfigurement [26]. Individuals having a low level of health literacy regarding essential information related to oral cancer, as it was found in this study, could lead to misinterpretations of early signs and symptoms of the disease. This misinterpretation could contribute to a delay in seeking a dentist’s advice, which may, in turn, lead to oral cancer only being detected at a more advanced stage. This effect of lacking oral cancer knowledge has also been reported in previous studies [17,27]. Therefore, knowledge of oral cancer was recognized as a modifiable determinant that aids in the early detection of oral cancer and might reduce oral cancer morbidity and mortality [28]. Our quantitative study revealed a substantial level of knowledge among the patient population. This could be attributed to the relatively high level of education of the participants in our quantitative study, as almost half of them had a university-level education. Despite the adequate knowledge of participants regarding oral cancer etiology, their knowledge of the local prevalence of oral cancer and its gender distribution was low.

Another determinant that may contribute to the early detection of oral cancer is the individual’s perception of the availability and accessibility of healthcare facilities. A negative perception of dental patients towards accessing a healthcare facility and the ability of healthcare workers to detect and treat oral cancer would adversely affect seeking a dentist’s advice. It would eventually lead to diagnosis at a later stage of the disease. The individual help-seeking behavior for oral cancer was also found to be influenced by access to healthcare, costs, competing responsibilities, work commitments, childcare, holidays and others [29]. The prevailing perception of the participants toward the availability and accessibility of healthcare facilities was negative.

Although examination for oral cancer causes feelings of fear and uncertainty among a substantial number of the surveyed patients, more than three quarters of the participants (both males and females) would prefer to receive oral cancer examinations in routine dental screening and education about oral cancer from their dentist. This can be considered a positive reflection of the participants caring about their health and their interest in improving their oral cancer awareness. A similar finding was also evident in a previous study, in which patients also favored being examined for oral cancer; however, in that study, dentists were concerned that their patients would be anxious because of the oral cancer examination [30]. In the present study, participants were not aware as to whether their dentists had examined them for oral cancer or not. Participants also mentioned that their dentists had not educated them about oral cancer. Moreover, participants declared their lack of knowledge and ability to perform oral cancer self-examinations.

A limitation of the present study was that all participants were from JDS clinics, and a relatively high percentage of the clinic visitors have a higher or university education. Hence, the external validity of the present study might be compromised. However, JDS clinics are the major dental clinics in the Jazan region of Saudi Arabia, receiving all patients with no restrictions on age, gender or nationality while providing all dental treatments free of cost. Additionally, the quantitative study’s data collection was planned to be completed before an international oral cancer conference in the Jazan region as the media attention regarding the conference may have had an external influence on the oral cancer awareness level of participants in the second study. Another limitation of the study is due to convenience sampling, where the responses may be biased. Additionally, due to the time constraint, all the etiological and risk factors could not be included in the study questionnaire.

## 5. Conclusions

JDS clinic patients have an adequate awareness of oral cancer and its risk factors. The practice of oral cancer examination and patient education of its risk factors is, however, limited. JDS clinic patients feel a need for oral cancer examinations to be embedded in routine dental practice.

## Figures and Tables

**Table 1:** Demographics of participating dental patients.

	*n* = 315	Mean	SD	Frequency	Percentage
**Age** (years)	290	* 31.0	11.1	-	-
**Sex**	313				
Male	-	-	-	129	41.2
Female	-	-	-	184	58.8
**Nationality**	249				
Saudi	-	-	-	214	85.9
Not Saudi	-	-	-	35	14.1
**Education Level**	315				
Uneducated	-	-	-	14	4.4
Primary	-	-	-	25	7.9
Intermediate	-	-	-	49	15.6
Secondary/High school	-	-	-	76	24.1
University	-	-	-	144	45.7
Higher education	-	-	-	7	2.2

* Mode = 25, range = (12–70).

**Table 2:** Dental patients’ knowledge toward oral cancer.

**a. Aetiology**		***n* = 315**	**Correct %**	**Correct**	**Not Correct**
1.	Oral cancer affects the oral cavity	309	57	176	133
2.	Oral cancer is a malignant disease	312	81.7	255	57
3.	Oral cancer may metastasize to the rest of the body	293	59	173	120
4.	Sores anywhere in or around your mouth are signs of oral cancer	306	64.1	196	110
5.	Swellings anywhere in or around your mouth are signs of oral cancer	305	33.4	102	203
6.	Lumps anywhere in or around your mouth are signs of oral cancer	309	38.8	120	189
7.	Thick patches anywhere in or around your mouth are signs of oral cancer	312	76.3	238	74
8.	Loose teeth without apparent dental cause is a sign of oral cancer	302	52	157	145
**b. Epidemiology**					
9.	Oral cancer is prevalent in Saudi Arabia	311	68.8	214	97
10.	The Jazan area has the highest prevalence of oral cancer in comparison with other areas in Saudi Arabia	310	34.8	108	202
11.	Oral cancer in Jazan is more prevalent among females than males	306	22.2	68	238
**c. Risk Factors**					
12.	Genes play a role in the pathogenesis of oral cancer	303	79.2	240	63
13.	Smoking is a causing factor of oral cancer	306	82	251	55
14.	Using ghat is not a causing factor of oral cancer	308	60.1	185	123
15.	Using shammah is a causing factor of oral cancer	313	77.6	243	70
**d. Preventive Measures**					
16.	Avoiding smoking/shammah prevents oral cancer	308	80.2	247	61
17.	Stopping smoking/shammah prevents oral cancer	306	76.8	235	71
18.	Sports prevent oral cancer	309	40.1	124	185
19.	Eating vegetables prevents oral cancer	314	83.8	263	51

**Table 3:** Dental patients’ perception toward oral cancer.

**a. Detection**		***n* = 315**	**Strongly Disagree**		**Disagree**		**Neither Agree nor Disagree**		**Agree**		**Strongly Agree**	
			**F**	**%**	**F**	**%**	**F**	**%**	**F**	**%**	**F**	**%**
1.	Oral self-examination is useful in detecting oral cancer	313	14	**4.5**	8	**2.6**	16	**5.1**	165	**52.7**	110	**35.1**
2.	Regular visits to the dentist would help early detection of oral cancer	309	14	**4.5**	4	**1.3**	10	**3.2**	136	**44**	145	**46.9**
3.	Oral examination is beneficial in detecting oral cancer	308	14	**4.5**	5	**1.6**	12	**3.9**	150	**48.7**	127	**41.2**
**b. Clinical Examination**												
4.	Oral cancer examinations must be a routine in dental clinics	305	14	**4.6**	21	**6.9**	43	**14.1**	132	**43.3**	95	**31.3**
5.	Dentists often skip oral cancer examinations due to lack of knowledge	306	20	**6.5**	69	**22.5**	112	**36.6**	77	**25.2**	28	**9.2**
6.	Dentists often skip oral cancer examinations because they do not care	308	44	**14.3**	91	**29.5**	86	**27.9**	59	**19.2**	28	**9.1**
7.	Dentists often skip oral cancer examinations because they only focus on patients’ complaints.	307	29	**9.4**	53	**17.3**	67	**21.8**	102	**33.2**	56	**18.2**
8.	Dentists often skip oral cancer examinations due to lack of time	309	28	**9.1**	68	**22**	98	**31.7**	92	**29.8**	23	**7.4**
9.	Gender of the patient is related to performing oral cancer examinations in the dental clinic	302	23	**7.6**	87	**28.8**	105	**34.8**	72	**23.8**	15	**5**
10.	Gender of the dentist is related to performing oral cancer examinations in the dental clinic	311	42	**13.5**	84	**27**	98	**31.5**	71	**22.8**	16	**5.1**
11.	Age of the dentist is related to performing oral cancer examinations in the dental clinic	303	44	**14.5**	99	**32.7**	71	**23.4**	76	**25.1**	13	**4.3**
12.	Nationality of the dentist is related to performing oral cancer examinations in the dental clinic	307	54	**17.6**	100	**32.6**	87	**28.3**	60	**19.5**	6	**2**
13.	Where the dentist meets the patient (hospital or dental clinic) is related to performing oral cancer examinations by the dentist	310	21	**6.8**	42	**13.5**	67	**21.6**	142	**45.8**	38	**12.3**
14.	If my dentist examined me for oral cancer, I would feel scared	312	31	**9.9**	66	**21.2**	55	**17.6**	119	**38.1**	41	**13.1**
15.	If my dentist examined me for oral cancer, I would feel comfortable	301	29	**9.6**	44	**14.6**	65	**21.6**	115	**38.2**	48	**15.9**
16.	If my dentist examined me for oral cancer, I would feel uncertain of the reasons for such an exam	301	22	**7.3**	53	**17.6**	84	**27.9**	102	**33.9**	40	**13.3**
**c. Preventive Measures**												
17.	Having health educators at dental clinics who address oral health will raise the awareness of oral cancer in the community	304	12	**3.9**	11	**3.6**	28	**9.2**	90	**29.6**	163	**53.6**
18.	Conducting symposiums about oral cancer for the community will raise the awareness of oral cancer in the community	309	10	**3.2**	12	**3.9**	30	**9.7**	95	**30.7**	162	**52.4**
19.	Conducting lectures about oral cancer for the community will raise the awareness of oral cancer in the community	310	16	**5.2**	13	**4.2**	24	**7.7**	98	**31.6**	159	**51.3**
20.	Distributing flyers about oral cancer for the community will raise the awareness of oral cancer in the community	311	9	**2.9**	16	**5.1**	24	**7.7**	115	**37**	147	**47.3**
21.	Banning ghat, shammah and tobacco will decrease the incidence of oral cancer in the community	312	18	**5.8**	10	**3.2**	40	**12.8**	70	**22.4**	174	**55.8**

**Table 4:** Dental patients’ practice toward oral cancer.

**a. Detection**		***n* = 315**	**Strongly Disagree**		**Disagree**		**Neither Agree or Disagree**		**Agree**		**Strongly Agree**	
			**F**	**%**	**F**	**%**	**F**	**%**	**F**	**%**	**F**	**%**
41.	My dentist did not examine me for oral cancer because I did not show any signs or symptoms of oral cancer	313	25	**8**	43	**13.7**	82	**26.2**	127	**40.6**	36	**11.5**
42.	My dentist did not examine me for oral cancer because oral cancer is not common in the Jazan region	312	49	**15.7**	102	**32.7**	89	**28.5**	54	**17.3**	18	**5.8**
43.	My dentist did not examine me for oral cancer because I visit him/her for a certain dental issue	310	22	**7.1**	57	**18.4**	68	**21.9**	135	**43.5**	28	**9**
44.	Oral cancer educational materials are not available in the dental college clinics	312	34	**10.9**	61	**19.6**	98	**31.4**	71	**22.8**	48	**15.4**
**b. Clinical Examination by Dentist**		***n*** **= 315**	**Never**		**Rarely**		**Some-times**		**Often**		**Always**	
			**F**	**%**	**F**	**%**	**F**	**%**	**F**	**%**	**F**	**%**
45.	My dentist examined me for oral cancer	313	195	**62.3**	44	**14.1**	48	**15.3**	17	**5.4**	9	**2.9**
46.	I would go to the dentist if I noticed mucosal changes in my mouth	299	20	**6.7**	36	**12**	67	**22.4**	83	**27.8**	93	**31.1**
47.	I would go to the dentist if I noticed sores in my mouth	309	22	**7.1**	27	**8.7**	61	**19.7**	70	**22.7**	129	**41.7**
48.	I would go to the dentist if I noticed swellings in my mouth	306	21	**6.9**	21	**6.9**	46	**15**	56	**18.3**	162	**52.9**
49.	I would go to the dentist if I noticed lumps in my mouth	310	21	**6.8**	27	**8.7**	37	**11.9**	62	**20**	163	**52.6**
50.	I would go to the dentist if I noticed thick patches in my mouth	303	19	**6.3**	23	**7.6**	49	**16.2**	61	**20.1**	151	**49.8**
**c. Clinical Education**												
51.	My dentist informed me about conducting an oral cancer examination on me	305	168	**55.1**	37	**12.1**	43	**14.1**	25	**8.2**	32	**10.5**
52.	My dentist educated me about oral cancer and its risk factors	310	152	**49**	57	**18.4**	35	**11.3**	35	**11.3**	31	**10**
**d. Self-Examination**												
53.	I do oral cancer self-examinations	312	186	**59.6**	48	**15.4**	27	**8.7**	28	**9**	23	**7.4**
**e. Perceived Dentist Role**		***n*** **= 315**	**Strongly Oppose**		**Somewhat Oppose**		**Neutral**		**Somewhat Favor**		**Strongly Favor**	
			**F**	**%**	**F**	**%**	**F**	**%**	**F**	**%**	**F**	**%**
54.	The dentist should educate patients about oral cancer in the clinic	311	10	**3.2**	9	**2.9**	21	**6.8**	44	**14.1**	227	**73**
55.	The dentist should refer patients to a tobacco-cessation program if needed	309	9	**2.9**	11	**3.6**	27	**8.7**	47	**15.2**	215	**69.6**

## Data Availability

The data presented in this study are available on request from the corresponding author. The data are not publicly available as they contain information that could compromise the privacy of the research participants.

## Supplementary Materials

The following are available online at https://www.mdpi.com/article/10.3390/ijerph18147562/s1, File S1: The coding process and interview guide.

## References

[1] Warnakulasuriya S. (2009). Global epidemiology of oral and oropharyngeal cancer. Oral Oncol..

[2] Saudi Cancer Registry Saudi Arabia Cancer Incidence Report 2012. http://ghdx.healthdata.org/organizations/saudi-cancer-registry.

[3] Saudi Cancer Registry Saudi Arabia Cancer Incidence Report 1999–2000. http://ghdx.healthdata.org/organizations/saudi-cancer-registry.

[4] Saudi Cancer Registry Saudi Arabia Cancer Incidence Report 2008. http://ghdx.healthdata.org/organizations/saudi-cancer-registry.

[5] Mashberg A., Samit A. (1995). Early diagnosis of asymptomatic oral and oropharyngeal squamous cancers. CA. Cancer J. Clin..

[6] Jan J.-C., Hsu W.-H., Liu S.-A., Wong Y.-K., Poon C.-K., Jiang R.-S., Jan J.-S., Chen I.-F. (2011). Prognostic factors in patients with buccal squamous cell carcinoma: 10-year experience. J. Oral Maxillofac. Surg..

[7] Kreppel M., Eich H.T., Kübler A., Zöller J.E., Scheer M. (2010). Prognostic value of the sixth edition of the UICC’s TNM classification and stage grouping for oral cancer. J. Surg. Oncol..

[8] Dissanayaka W.L., Pitiyage G., Kumarasiri P.V.R., Liyanage R.L.P.R., Dias K.D., Tilakaratne W.M. (2012). Clinical and histopathologic parameters in survival of oral squamous cell carcinoma. Oral Surg. Oral Med. Oral Pathol. Oral Radiol..

[9] Gómez I., Seoane J., Varela-Centelles P., Diz P., Takkouche B. (2009). Is diagnostic delay related to advanced-stage oral cancer? A meta-analysis. Eur. J. Oral Sci..

[10] Asthana S., Labani S., Kailash U., Sinha D.N., Mehrotra R. (2019). Association of Smokeless Tobacco Use and Oral Cancer: A Systematic Global Review and Meta-Analysis. Nicotine Tob. Res..

[11] Warnakulasuriya S. (2004). Smokeless tobacco and oral cancer. Oral Dis..

[12] Chang M.-C., Lin L.-D., Wu H.-L., Ho Y.-S., Hsien H.-C., Wang T.-M., Jeng P.-Y., Cheng R.-H., Hahn L.-J., Jeng J.-H. (2013). Areca nut-induced buccal mucosa fibroblast contraction and its signaling: A potential role in oral submucous fibrosis—A precancer condition. Carcinogenesis.

[13] Idris A.M., Nair J., Friesen M., Ohshima H., Brouet I., Faustman E.M., Bartsch H. (1992). Carcinogenic tobacco-specific nitrosamines are present at unusually high levels in the saliva of oral snuff users in Sudan. Carcinogenesis.

[14] Zhang X., Schmitz W., Gelderblom H.R., Reichart P.A. (2001). Shammah-induced oral leukoplakia-like lesions. Oral Oncol..

[15] Allard W.F., DeVol E.B., Te O.B. (1999). Smokeless tobacco (shamma) and oral cancer in Saudi Arabia. Community Dent. Oral Epidemiol..

[16] de Vries H. (2017). An Integrated Approach for Understanding Health Behavior; The I-Change Model as an Example. Psychol. Behav. Sci. Int. J..

[17] Warnakulasuriya K.A., Harris C.K., Scarrott D.M., Watt R., Gelbier S., Peters T.J., Johnson N.W. (1999). An alarming lack of public awareness towards oral cancer. Br. Dent. J..

[18] Grant E., Silver K., Bauld L., Day R., Warnakulasuriya S. (2010). The experiences of young oral cancer patients in Scotland: Symptom recognition and delays in seeking professional help. Br. Dent. J..

[19] Andersen B.L., Cacioppo J.T., Roberts D.C. (1995). Delay in seeking a cancer diagnosis: Delay stages and psychophysiological comparison processes. Br. J. Soc. Psychol..

[20] Mitchell E., Macdonald S., Campbell N.C., Weller D., Macleod U. (2008). Influences on pre-hospital delay in the diagnosis of colorectal cancer: A systematic review. Br. J. Cancer.

[21] Warnakulasuriya K.A., Johnson N.W. (1999). Dentists and oral cancer prevention in the UK: Opinions, attitudes and practices to screening for mucosal lesions and to counselling patients on tobacco and alcohol use: Baseline data from 1991. Oral Dis..

[22] Jafer M., Crutzen R., Moafa I., van den Borne B. (2021). What Do Dentists and Dental Students Think of Oral Cancer and Its Control and Prevention Strategies? A Qualitative Study in Jazan Dental School. J. Cancer Educ..

[23] Jafer M., Crutzen R., Halboub E., Moafa I., van den Borne B., Bajonaid A., Jafer A., Hedad I. (2020). Dentists Behavioral Factors Influencing Early Detection of Oral Cancer: Direct Clinical Observational Study. J. Cancer Educ..

[24] Tong A., Sainsbury P., Craig J. (2007). Consolidated criteria for reporting qualitative research (COREQ): A 32-item checklist for interviews and focus groups. Int. J. Qual. Health Care.

[25] Chapman A.L., Hadfield M., Chapman C.J. (2015). Qualitative research in healthcare: An introduction to grounded theory using thematic analysis. J. R. Coll. Physicians Edinb..

[26] Shimpi N., Jethwani M., Bharatkumar A., Chyou P.-H., Glurich I., Acharya A. (2018). Patient awareness/knowledge towards oral cancer: A cross-sectional survey. BMC Oral Health.

[27] Horowitz A.M., Nourjah P., Gift H.C. (1995). U.S. adult knowledge of risk factors and signs of oral cancers: 1990. J. Am. Dent. Assoc..

[28] Villa A., Kreimer A.R., Pasi M., Polimeni A., Cicciù D., Strohmenger L., Gherlone E., Abati S. (2011). Oral cancer knowledge: A survey administered to patients in dental departments at large Italian hospitals. J. Cancer Educ..

[29] Scott S.E., Grunfeld E.A., Auyeung V., McGurk M. (2009). Barriers and triggers to seeking help for potentially malignant oral symptoms: Implications for interventions. J. Public Health Dent..

[30] Choi Y., Dodd V., Watson J., Tomar S.L., Logan H.L., Edwards H. (2008). Perspectives of African Americans and dentists concerning dentist-patient communication on oral cancer screening. Patient Educ. Couns..

