# Supplementary material for: Using the Exploratory Sequential Mixed Methods Design to Investigate Dental Patients’ Perceptions and Needs Concerning Oral Cancer Information, Examination, Prevention and Behavior
Source: Int J Environ Res Public Health. 2021 Jul 16;18(14):7562. doi: 10.3390/ijerph18147562 (PMC8307210; doi:10.3390/ijerph18147562)
Supplement: Supplementary file 1 [file ijerph-18-07562-s001.zip › ijerph-1233249-supplementary.pdf]

Coding Process Table

Responses									
"I have no idea; a tumor may be a tumor" X4	"I don't know" X17	"I have no idea" X12	"Regular check-ups" X7	"Not available in Jazan area" X24	"it is useful, it helps early detection of the cancer" X24	"I have no perception of that" X11	"I don't think my dentist ever examined me" X4	"No, he has not" X36	"I do regular self-check-ups, it is a good experience"
"It is a tumor in the mouth, or the oral cavity" X23	"The area is not endemic with Oral cancer, because I don't have any complaints" X2	"Smoking, Ghat and Shammah" X17	"I am not well aware of them" X9	"It is available but hard to access"	"No, I don't think routine clinical examination will be useful"	"it would be helpful if dentists would educate their patients about oral cancer" X29	"No, because I didn't ask him, and maybe because I don't have symptoms"	"My dentist educated me about oral cancer and it was informative" X4	"I don't do self-examination because I don't know how to do so". X39
"Ulcers or wounds inside the mouth of a person who smokes or uses Ghat that turns into cancer"	"I never heard that the area is endemic" X3	"Smoking or any facilitator of bacteria" X2	"by maintaining oral hygiene" X9	"It is available in the area" X7	"it is useful, it helps early detection of the cancer, but I would be scared to be on the		"No, because it is not widespread"		

					receiving end" X15				
"Ulcers or stains in the mouth that are cancerous" X2	"Yes, the area is endemic with oral cancer" X17	"Smoking and carelessness" X2	"No smoking and good oral hygiene" X10	"I don’t know" X8			"No, he has not examined me" X31		
"It is an infection due to intake of expired food"	"No, the area is not endemic"	"Genetic mutations give rise to oral cancer"	"I have no idea" X2				"Yes, my dentist did an oral examination on me" X3		
"I have no idea what this disease is" X6		"Environment"							
"it is a fungal infection that was neglected becomes cancer" X2		"Alcohol and smoking" X2	"Sunlight prevents against oral cancer"						
"It is due to an infection"		"poor oral hygiene" X2							

		"Other cancers cause oral cancer"							
**Initial Codes**									
Etiology	Epidemiology	Risk Factors	Prevention	Detection	Clinical examination	Preventive methods	Clinical examination	Clinical Education	Self-examination
**Focus Codes**									
Knowledge				Perception			Practice		
**Theoretical Codes**									
1) JDS-clinic patients have low awareness of oral cancer and its risk factors<br>2) The practice of oral cancer examination and patient education on its risk factors is limited<br>3) JDS-clinic patients feel that there is a need for more attention towards oral cancer examinations									

Appendix A

JDS Patients Interview Guide

Questions:

1) What do you know about oral cancer?

2) In your opinion, how could individual develop oral cancer?

  - What do you think are the risk factors of oral cancer?

  - How it can be prevented?

3) Do you think that the cancer of oral cavity is an issue in Jazan?

  - If yes how /why? How serious is the problem?

  - If no: why?

4) Do you think you or any one member of your family/community is at risk of oral cancer?

  - If yes how /why?

  - If no: why?

5) What do you think about oral cancer examination/ screening?

  - In your opinion, do you think it is useful?

    - If yes how /why?

    - If no: why?

6) What do you feel toward performing an oral cancer examination/ screening for you in the dental clinic?

7) Have you had an oral cancer examination done to you in dental or other clinic?

- If yes: what were your experiences with this?
- If no: why think dentists didn’t do so? Do you think you can do anything about it?

8) Do you think that oral cancer examination/ screening is accessible in Jazan?

- If no: why? Do you think you can do anything about it?

9) Do you think that oral cancer treatment is possible in Jazan?

- If no: why? Do you think you can do anything about it?

10) Have you had any education on oral cancer and their risks while in dental or other clinic?

- If yes: what were your experiences with this?
- If no: why think dentists didn’t do so?

11) What do you feel toward educating patients of oral cancer during dental clinic examination/ screening?

12) In your opinion, do you think that there are any/enough oral cancer education materials in Jazan dental school clinics?

- If yes: do you think it is followed? If not: why?
- If no: why?

13) Do you currently engage in oral cancer self-examination/ screening?

- If yes: what were your experiences with this?
- If no: why?

14) Do you have any thing that you would like to add?
